# Decoding mitochondrial stress genes in DCM: towards precision diagnosis and therapy

**DOI:** 10.1186/s41065-025-00399-3

**Published:** 2025-04-11

**Authors:** Bingbing Zhu, Hai Cheng, Jiawei Li, Yangyang Hu, Xiaoning Ge

**Affiliations:** 1 Department of Cardiology, Suzhou Kowloon Hospital, Shanghai Jiaotong University School of Medicine, Suzhou, Jiangsu Province 215028 China; 2 Department of Rheumatology and Immunology, Tongji Hospital, Tongji Medical College, Huazhong University of Science and Technology, Wuhan, Hubei Province 430030 China

**Keywords:** Dilated cardiomyopathy, Immune infiltration, Diagnosis, Oxidative stress, Bioinformatics, Mitochondria

## Abstract

**Background:**

Mitochondrial oxidative stress (ROS) is a crucial factor in the pathogenesis of dilated cardiomyopathy (DCM). Despite its significance, robust biomarkers for assessing its role remain scarce. This study investigates ROS mechanisms in DCM and identifies associated biomarkers, offering fresh insights into diagnosis and treatment.

**Methods:**

We sourced transcriptomic data from the GEO database and mitochondrial oxidative stress-related genes from GeneCards. Using consensus clustering, we identified 64 genes associated with mitochondrial oxidative stress in DCM and further isolated five hub genes through protein–protein interaction and machine learning techniques. These genes were analyzed for functions related to immunity, drug sensitivity, and single-cell localization. Concurrently, we collected blood samples from DCM patients to validate the hub genes' expression.

**Results:**

The study identified five hub genes related to mitochondrial oxidative stress: VCL, ABCB1, JAK2, KDR, and NGF. Expression analysis revealed high levels of VCL, ABCB1, KDR, and NGF in the non-failing (NF) group, while JAK2 was elevated in the DCM group (*p* < 0.05). Diagnostic efficacy, measured by area under the curve (AUC), was significant for VCL (76.4), ABCB1 (80.1), JAK2 (68.2), KDR (78.1), and NGF (71.8). Moreover, several drugs were identified as potential regulators of these hub genes, including Topotecan, CDK9_5576, Acetalax, Afatinib, and GSK591. Notably, VCL showed increased expression in DCM patient blood samples, consistent with transcriptomic and single-cell findings.

**Conclusion:**

This research highlights key genes associated with mitochondrial oxidative stress—VCL, ABCB1, JAK2, KDR, NGF—that show differential expression in DCM and myocardial infarction. These findings underscore their diagnostic potential and pave the way for new therapeutic strategies.

## Introduction

Dilated cardiomyopathy (DCM) is characterized by left ventricular or biventricular enlargement and systolic dysfunction. As a common clinical entity, DCM poses significant treatment challenges and generally carries a poor prognosis. Treatment modalities for DCM currently include conventional pharmacological therapies, primarily beta blockers, diuretics, and cardiac stimulants, as well as non-pharmacological interventions such as surgical procedures, emerging immunotherapies, stem cell transplantation, and gene therapy [1–5]. DCM is a leading cause of sudden cardiac death (SCD) and heart failure (HF) and remains the predominant reason for heart transplantation among children and adults globally [2]. Various etiologies, including genetic, infectious, and inflammatory conditions, contribute to DCM [3]. Its prevalence in the general population ranges from 1:250 to 1:2500 [4, 5]. Without prompt intervention, the one-year survival rate for DCM patients is 70–75%, decreasing to around 50% over five years. Recent research has shed light on the genetic underpinnings of both hereditary and idiopathic DCM, revealing a higher incidence of mutations in genes associated with structural proteins of the cytoskeleton, cardiomyocyte sarcomere, and nuclear membrane [6–8]. The surge in gene detection for cardiovascular diseases in recent years shows a detection rate of about 60–70% for hypertrophic cardiomyopathy but less than 60% for DCM [9, 10]. This underscores the urgent need to identify novel biomolecular markers that correlate significantly with DCM to enhance the efficacy and timeliness of diagnostic and therapeutic strategies.

Mitochondria serve as the primary sources of reactive oxygen species (ROS) in cells. Under physiological conditions, ROS are involved in cellular signaling; however, excessive ROS lead to oxidative stress, damaging mitochondrial DNA, proteins, and lipids [11]. In dilated cardiomyopathy (DCM), mitochondrial dysfunction disrupts energy metabolism and the increased ROS production exacerbates damage to cardiomyocytes [12]. Various mechanisms [13] through which oxidative stress influences the progression of DCM include: firstly, ROS can directly impair myocardial cell membranes, reducing their fluidity and integrity; secondly, oxidative stress can trigger inflammatory pathways and promote the release of cytokines, intensifying the myocardial inflammatory response [14]. Additionally, ROS may induce cardiomyocyte apoptosis by interfering with apoptosis-related signaling pathways. Mitochondrial oxidative stress is also linked to the dysregulation of calcium ions (Ca^2^⁺) in DCM. Mitochondria are crucial for maintaining the balance of intracellular calcium ions. Oxidative stress can impair mitochondrial function, responsible for calcium ion regulation in myocardial cells, leading to calcium imbalances that adversely affect the contraction and relaxation of cardiac muscle [15, 16].

In this research, two GEO datasets were utilized to identify significant differentially expressed genes (DEGs) between normal and dilated cardiomyopathy (DCM) samples. To reduce redundancy among the genes, both LASSO tenfold cross-validation and random forest algorithms were applied. Subsequently, five potential genes were selected for detailed analysis. Analytical methods, including ROC analysis, differential expression analysis, and RT-qPCR, were conducted on these five genes. The findings indicate that these genes have promising predictive capabilities.

## Materials and methods

### Data acquisition

The clinical and transcriptomic data for individuals with Dilated Cardiomyopathy (DCM) were sourced from the GEO database (GEO, http://www.ncbi.nlm.nih.gov/geo). We established clear inclusion and exclusion criteria for case selection: Inclusion Criteria: Samples must originate from the left ventricle and represent either NF (Non-Failing) or DCM types; datasets must include at least 35 pathological samples and over 3000 cells from single-cell specimens. Exclusion Criteria: Any samples not classified as NF or DCM or those lacking comprehensive clinical and pathological details were excluded. Our analysis encompassed a total of 383 qualifying samples, including 51 from the GSE116250 dataset—14 NF and 37 DCM left ventricular tissue samples [17, 18]. Another 332 samples were from the GSE141910 dataset, evenly divided between NF and DCM types. Importantly, the study strictly involved NF and DCM samples, excluding all other types [19]. Transcriptomic analyses utilized FPKM for downstream processing. Single-cell data specific to DCM were sourced from GSE121893, which included eight samples, with two controls and four DCM samples. This subset incorporated a total of 21,422 cells [20]. Pre-quality control measures, standardization, and batch effect mitigation were conducted using Seurat V5 software [21]. For gene selection related to mitochondria and oxidative stress, we employed the Genecards database (https://www.genecards.org/). Here, a search for 'mitochondria' yielded 11,827 genes, from which 2,933 were identified as mitochondria-related using a relevance score above 1. Similarly, 'oxidative stress' was used to find 13,632 genes, with 2,068 meeting the relevance score threshold of over 5 for inclusion [22].

### Protein–protein network interaction (PPI)

The STRING database (https://string-db.org/) was utilized to identify and predict protein–protein interactions (PPIs) [23]. This comprehensive resource amalgamates known and predicted PPIs derived from a variety of sources, including experimental results, computational predictions, and curated databases. Its extensive integration capabilities make it an indispensable tool for constructing and analyzing PPI networks in biological research. Following the identification of PPIs, the Cytoscape software platform is employed for visualization. Cytoscape is renowned for its ability to effectively display complex networks and integrate them with various types of attribute data. To further analyze these networks, the MCODE algorithm within Cytoscape is used to isolate relevant subnetworks [24]. MCODE excels at detecting densely connected regions within the PPI network, which are often indicative of functional modules or protein complexes.

### Variation analysis

This study utilized the Limma package (Linear Model for Microarray Analysis) for identifying and conducting statistical analyses of differentially expressed genes [25]. The Limma package is a highly regarded R package designed for gene expression data analysis, employing empirical Bayesian methods to enhance the precision and reliability of its statistical outputs. Initially, the raw gene expression data underwent background correction and normalization to mitigate technical variations that may have occurred between experimental batches. We then applied Limma’s lmFit function to fit a linear model to each sample, accounting for biological variations and experimental conditions. The parameters of this model were refined using an empirical Bayes function, which helps in achieving more accurate estimates of gene expression differences. Upon fitting the model, Limma's statistical methods were used to pinpoint genes with differential expression. Genes showing a log-fold change (logFC) greater than 0.5 and an adjusted *p*-value below 0.05 were identified as significantly differentially expressed.

### Enrichment analysis

There are two commonly used gene enrichment analysis methods: ORA and GSEA. ORA (Over-Representation Analysis) is employed to determine whether specific gene sets or functional categories are significantly overrepresented in a selected set of genes compared to a random distribution. This method is frequently applied in Gene Ontology (GO) analysis and KEGG pathway enrichment analysis. GSEA (Gene Set Enrichment Analysis), in contrast, not only identifies differentially expressed genes but also considers the direction and consistency of their expression trends. GSEA evaluates whether a specific gene set is significantly enriched within a ranked gene list by calculating an Enrichment Score (ES), which reflects the distribution pattern of genes from a given set within the overall expression dataset. GO and KEGG are among the most widely utilized databases for enrichment analysis in bioinformatics. In this study, to identify differentially expressed genes associated with relevant pathways, a Gene Ontology (GO) enrichment analysis was performed using the clusterProfiler package in R for a selected set of differentially expressed genes. Network plots were generated using the enrichplot package [26], and Gene Set Variation Analysis (GSVA) was conducted using the GSVA package to assess the heterogeneity of various biological processes [27].

### Immunoinfiltration analysis

To assess the degree of immune infiltration, we employed the CIBERSORT algorithm, which is integrated into the IOBR package [28] and was used for immune infiltration analysis in this study. The CIBERSORT algorithm was selected due to its extensive validation and reliability in deconvoluting immune cell compositions from bulk RNA-seq data, enabling the quantification of various immune cell subsets in complex tissue samples. Each computational approach applies a distinct strategy based on gene expression characteristics to estimate the abundance of different immune cell populations [29]. By calculating the enrichment or relative abundance of marker genes, we accurately estimated the proportions of immune cell types in DCM and HF samples. This analysis was conducted to elucidate the immune landscape of these samples, which is essential for understanding the underlying immunological mechanisms and identifying potential therapeutic targets for immune-related diseases.

### Drug sensitivity analysis

Utilizing small molecule drug data from the GDSC database, we employed the R package “oncopredict” to predict drug sensitivity for each tumor sample [30]. The GDSC database was selected due to its comprehensive and high-quality drug sensitivity data, which includes gene expression profiles and IC50 values across a diverse range of cancer cell lines. This extensive dataset enables the development of robust predictive models for drug response. To estimate the half-maximal inhibitory concentration (IC50) for each drug, regression algorithms were applied, modeling the response based on DCM samples. The predicted IC50 values for each drug were then outputted. The purpose of this analysis is to identify potential drug candidates and assess their efficacy in treating specific tumor types. By estimating IC50 values, we can gain valuable insights into the sensitivity of different tumor samples to various drugs, thereby facilitating the selection of effective therapeutic agents for personalized cancer treatment.

### RNA extraction and real-time quantitative PCR (RT-qPCR)

Blood samples were collected from 12 patients diagnosed with DCM and from a control group of normal individuals. Key gene expression studies were performed using RT-qPCR. The study was approved by the Ethics Committee of the Suzhou Kowloon Hospital, Shanghai Jiao Tong University School of Medicine, and was conducted in accordance with the Declaration of Helsinki. RNA was extracted from blood using Trizol (Invitrogen, Carlsbad, CA, USA). The quantity and quality of the RNA were determined using a spectrophotometer (NanoDrop-2000, Thermo Fisher Scientific). The primer sequences employed in the study are shown in Table 1. The 2-^ΔΔ^Ct method was used to calculate RNA expression, and the Student's t-test was applied to compare the expression levels of each RNA between groups.

**Table 1:** The PCR primers

Primers	Sequences (5'–3')
VCL Forward	GGCTTCCCTTGATCTGACT
VCL Reverse	CGGAATCTGGGTGCAGTT
ABCB1 Forward	ATGCTCTTGACAGGTTCCA
ABDB1 Reverse	TGCGGGAAGCGTGATAC
JAK2 Forward	GCACCTCCACTCCATCC
JAK2 Reverse	GAGCCTGCGCCCTTCTA
KDR Forward	GGATGAGGAAGATGGAGAGA
KDR Reverse	GCTTGTTATGCCACTTGATG
NGF Forward	GCCATCACGCCACAGTTTC
NGF Reverse	ACAACTTTGGTATCGTGGAAGG
GAPDH Forward	TCGGAGTCAACGGATTTGGT
GAPDH Reverse	TTCCCGTTCTCAGCCTTGAC

### Statistic analysis

Statistical analyses were performed to assess the significance of the differences and correlations observed in the study. All data are expressed as mean ± standard deviation (SD). Pearson correlation analysis was employed to explore relationships between variables. Statistical analysis and scientific plotting were conducted using R Studio (version 4.3.2), with a significance level of *p* < 0.05 considered statistically significant.

## Results

### Identification of differential oxidative stress genes in DCM

**Fig. 1:**
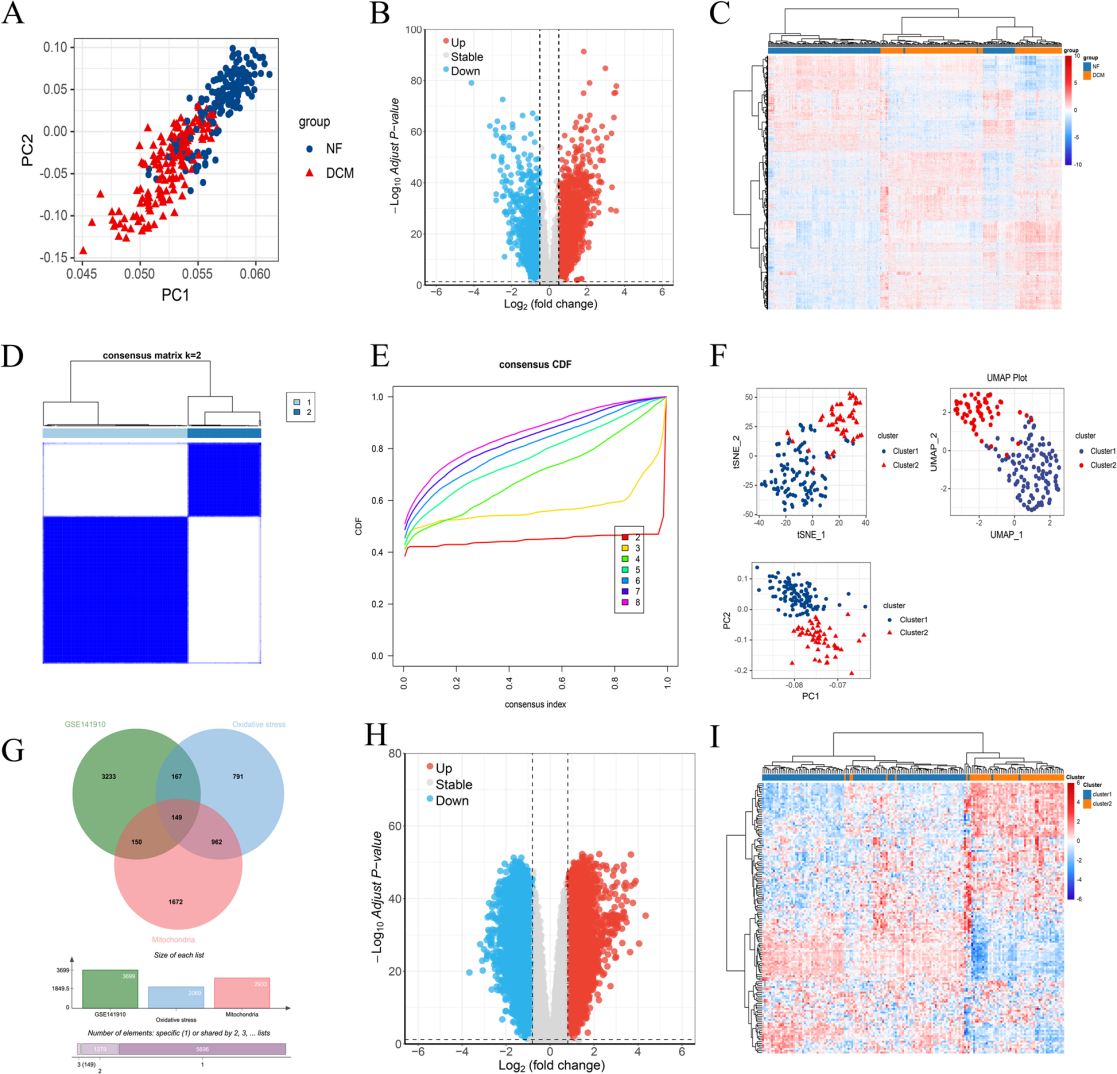
Identification of differential oxidative stress genes in DCM. **A** NF and DCM were subjected to a reduction in dimension through principal component analysis. **B** Differential gene volcanoes in GSE141910. **C** Difference analysis heat map in GSE141910. **D** Consistency clustering results for K = 2. **E** Consistency clustering CDF of K = 2 to 8. **F** Three-dimensionality reduction results (UMAP, TSNE and PCA) demonstrate differences in sample clustering. **G** Wayne diagram of the intersection of mitochondrial oxidation genes and differential genes. **H** Cluster1 and cluster2 difference gene volcano map. **I** Cluster1 and Cluster2 differential gene heat maps

A principal component analysis (PCA) was performed to reduce data dimensionality and facilitate clustering of 166 left ventricular tissue samples from the GSE141910 dataset, which included specimens from patients with non-failing (NF) and dilated cardiomyopathy (DCM) hearts. As shown in Fig. 1A, the results reveal significant differences between the two groups. Differential analysis was conducted, and the resulting volcano plots and accompanying heat maps are presented in Fig. 1B–C. Using a cutoff of |logFC|< 0.5, a total of 3755 differential genes were identified, comprising 2335 up-regulated and 1420 down-regulated genes. Given the heterogeneity of DCM among patients, consistent clustering was applied to the DCM samples in this dataset. The cumulative distribution function (CDF) curve indicates that the cluster obtained at K = 2 is the most stable (Fig. 1E), which is corroborated by the consistent clustering results (Fig. 1D). Three-dimensional reduction clustering analyses were subsequently carried out on both cluster 1 and cluster 2 samples using UMAP, t-SNE, and PCA; the results, displayed in Fig. 1F, further confirm significant differences between the two clusters. A subsequent differential analysis between the groups yielded a correlation matrix, with the corresponding heat map and volcano plot presented in Fig. 1H–I. In total, 6821 differential genes were identified, including 5678 up-regulated and 1143 down-regulated genes, using a logFC threshold of < 0.5. Additionally, mitochondrial and oxidative stress-related genes were extracted from Genecards and intersected with the previously identified differential genes. The resulting integration, depicted in Fig. 1G, yielded 149 DCM differential oxidative stress genes.

### Enrichment analysis

**Fig. 2:**
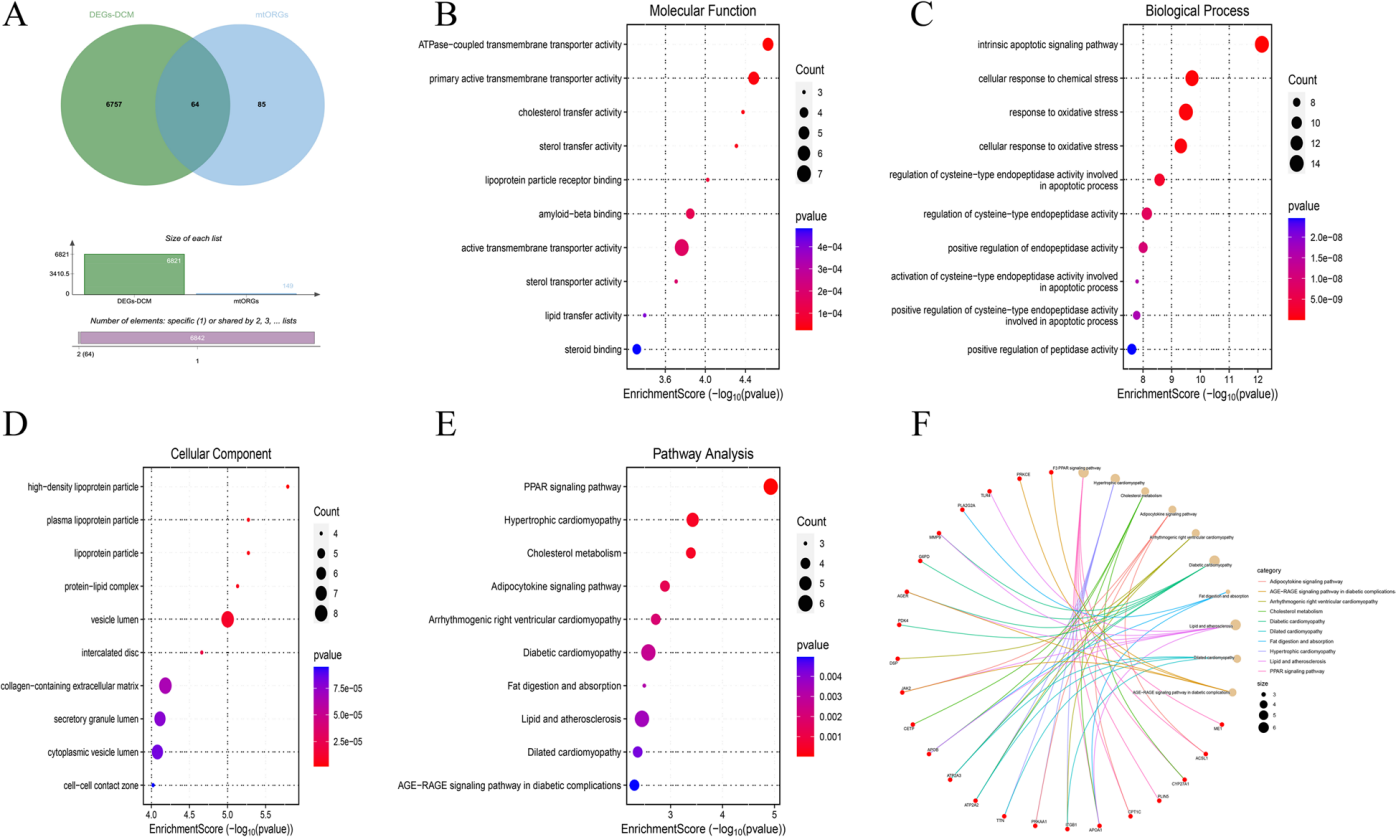
Intersection Gene Enrichment Analysis. **A** Wayne diagram of the intersection of different mitochondrial oxidation genes and different cluster difference genes. **B** Intersection gene GO enrichment molecular function bubble diagram. **C** Intersection gene GO enrichment biological process bubble diagram. **D** Intersection gene GO enrichment analysis cell composition bubble diagram. **E** Bubble plot of intersection gene GSEA enrichment analysis. **F** GO enrichment analysis network diagram

Figure 2A illustrates the intersection between the DCM differential oxidative stress genes and the differential genes identified between clusters, revealing a total of 64 overlapping genes. A subsequent Gene Ontology (GO) pathway enrichment analysis was conducted on these intersection genes. Regarding molecular function, the intersection genes were predominantly enriched in pathways associated with substance transport, including cholesterol transfer activity, lipid transfer activity, lipoprotein particle receptor binding, and ATPase-coupled transmembrane transporter activity (Fig. 2B). In terms of biological processes, these genes were mainly enriched in pathways related to oxidative stress and cell death (Fig. 2C). At the cellular component level, the genes were primarily localized to lipoprotein particles, cell–cell contact regions, and vesicle chambers (Fig. 2D). Gene set enrichment analysis (GSEA) further demonstrated that the intersection genes play a pivotal role in the pathogenesis of DCM, predominantly via the activation of the PPAR signalling pathway, cholesterol metabolism, the adipocytokine signalling pathway, and the AGE-SYS signalling pathway (Fig. 2E). Moreover, the network diagram of key pathways involved in the GO enrichment analysis revealed that the PPAR signalling pathway, the AGE-SYS signalling pathway, and lipid metabolism were more significantly enriched compared to other pathways (Fig. 2F).

### Joint machine learning to further screen hub genes

**Fig. 3:**
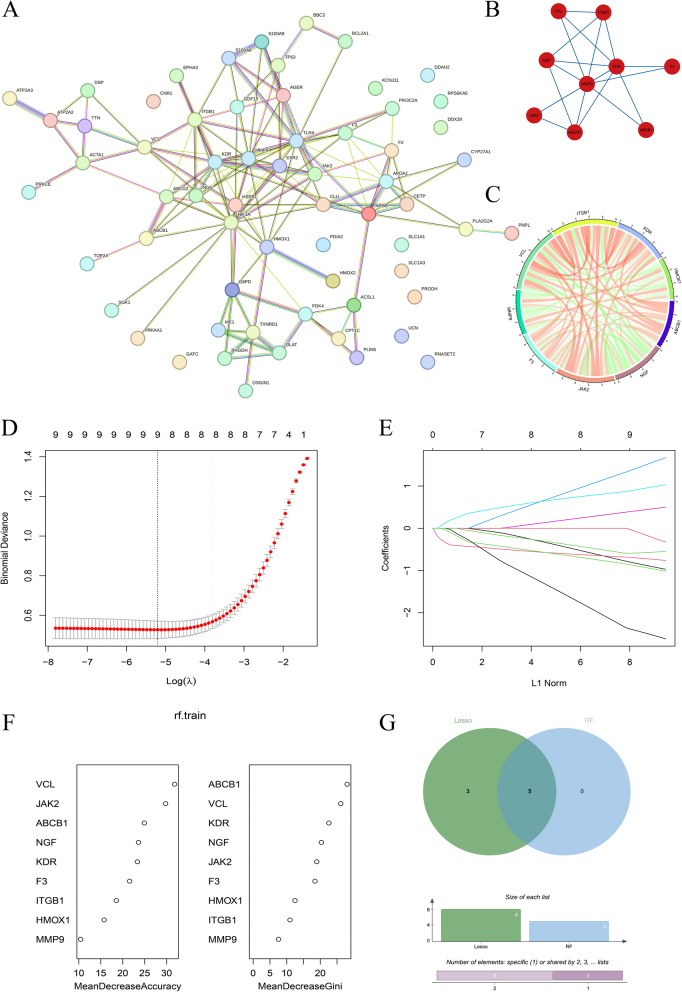
Multiple machine learning to further screen key genes. **A** Protein interaction network diagram of the intersection gene string in string database. **B** MCODE algorithm obtains protein interaction subnetworks. **C** Chord diagram of gene correlation of the subnetwork. **D** LASSO regression coefficient screening plot. **E** LASSO regression gene locus plot. **F** Random forest algorithm gene importance ranking. **G** LASSO gene and random forest gene intersection Venn diagram

A protein–protein interaction (PPI) network analysis was performed on the 64 intersecting genes, with the results presented in Fig. 3A. The MCODE algorithm was then applied to infer sub-networks, as shown in Fig. 3B. This analysis identified a total of nine sub-network genes, namely VCL, ITGB1, NGF, KDR, F3, MMP9, JAK2, HMOX1, and ABCB1. Notably, these nine PPI genes exhibited robust interaction relationships with other genes. The expression correlation analysis of the sub-network genes, depicted in Fig. 3C, revealed a strong positive correlation between ABCB1, JAK2, F3, ITGB1, and KDR with other genes, whereas HMOX1, NGF, and MMP9 displayed a strong negative correlation with other genes. To further refine the selection of sub-network genes, variable screening was conducted using both LASSO and random forest models. The LASSO results, presented in Fig. 3D and E, identified eight characteristic genes. Meanwhile, the random forest analysis (Fig. 3F) ranked the top five genes in terms of variable importance. The intersection of these two approaches resulted in the identification of five hub genes: VCL, ABCB1, JAK2, KDR, and NGF (Fig. 3G).

### Immunoinfiltration analysis

**Fig. 4:**
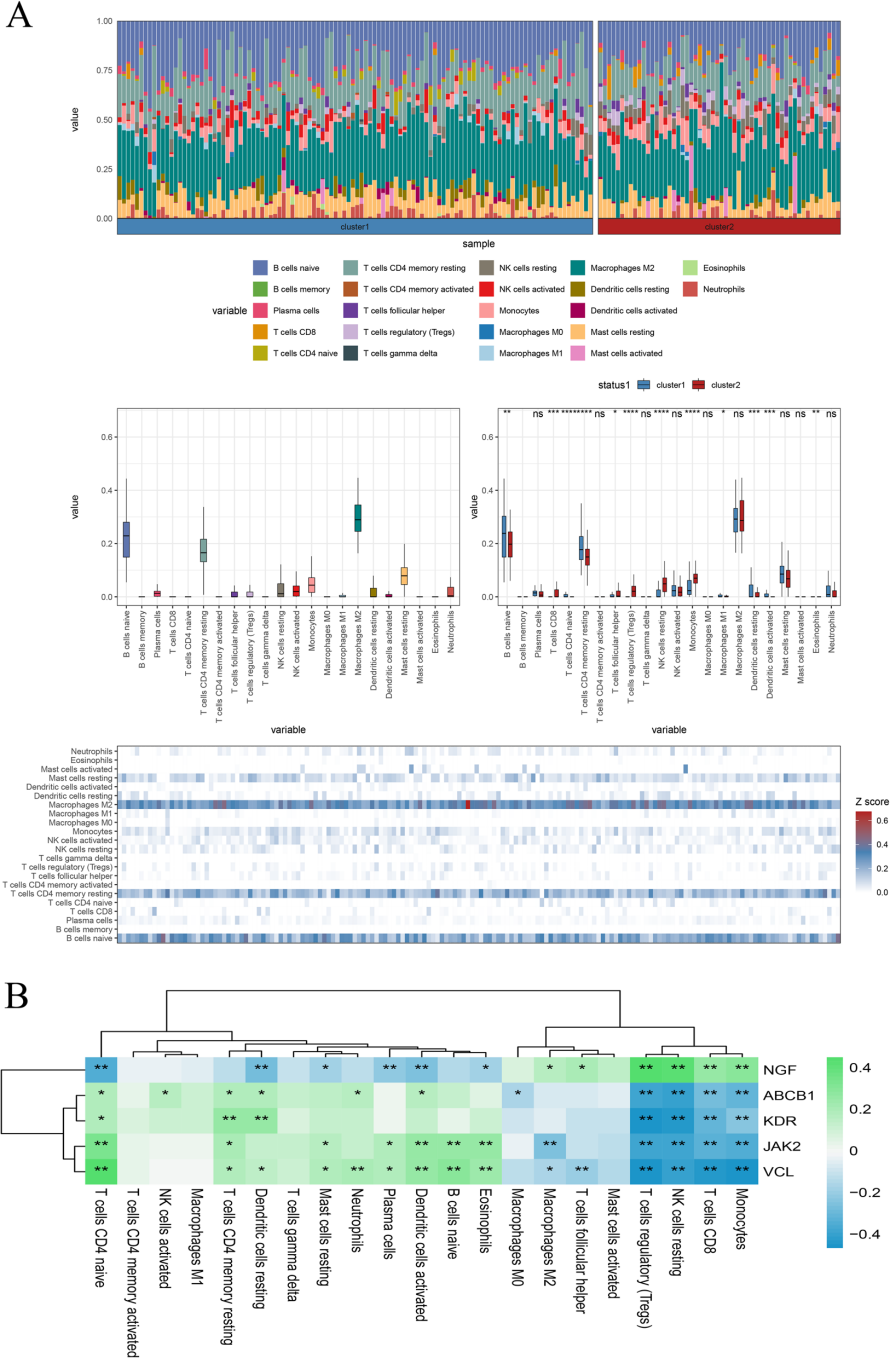
Immunoinfiltration analysis in GSE141910 dataset. **A** Difference of immune cells between DCM and NF group. **B** Heat map of correlation between Hub gene and immune cells in DCM samples

To determine differences in immune cell infiltration between the NF and DCM groups, the CIBERSORT algorithm was employed to assess the immune cell composition of the samples. The results, presented in Fig. 4A, indicate a greater infiltration of naive B cells, M2 macrophages, resting CD4 memory T cells, and resting mast cells in one of the groups. It is noteworthy that resting immune cells—specifically naive B cells, plasma cells, M2 macrophages, and dendritic cells—exhibited lower levels of infiltration, while T cells showed higher infiltration levels. Furthermore, a broad correlation between the hub genes and immune cell infiltration in DCM samples was observed (Fig. 4B). There was a significant negative correlation between the expression of ABCB1, KDR, JAK2, and VCL and the infiltration of monocytes, CD8 T cells, NK cells, and Tregs in DCM samples. Conversely, a significant positive correlation was found between the expression of CD4 naive T cells and resting CD4 memory T cells. Notably, the correlation pattern for the remaining four genes was opposite to that observed for NGF, suggesting potential differences in their functions.

### Differential expression of hub genes

**Fig. 5:**
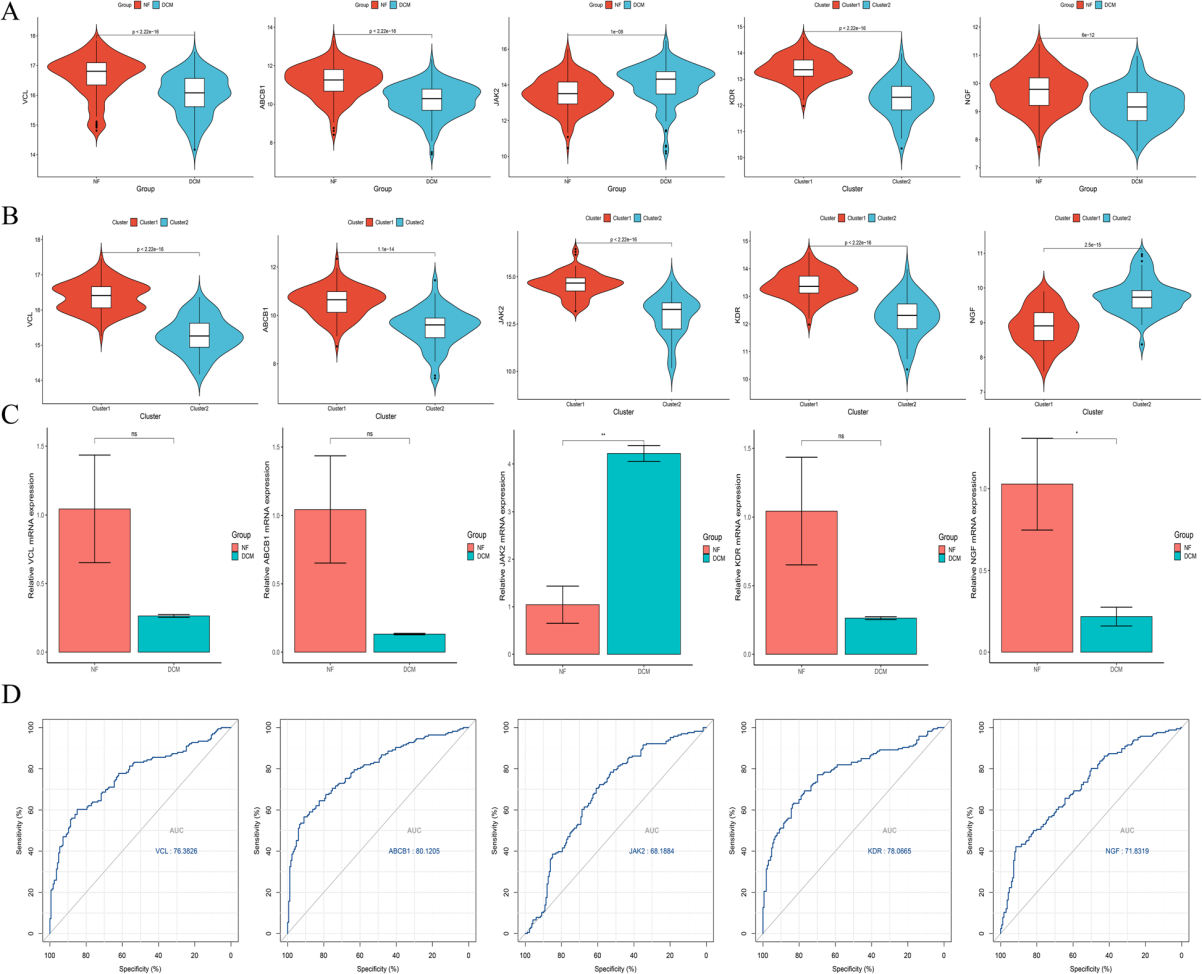
Differential expression of key genes. **A** Differential expression of key genes between NF and DCM groups. **B** Differential expression of key genes between cluster 1 and cluster 2. **C** RT-qPCR results for key genes. **D** ROC curve of key genes in predicting disease or not in a sample

The expression trends of the hub genes between DCM and NF samples were evaluated, as shown in Fig. 5A. It was observed that VCL, ABCB1, KDR, and NGF were highly expressed in the NF group, whereas JAK2 was predominantly expressed in the DCM group (*p* > 0.05). Moreover, the expression levels of VCL, ABCB1, JAK2, and KDR were markedly elevated in cluster 1 compared to cluster 2, while NGF exhibited higher expression in cluster 2 (Fig. 5B). The RT-qPCR results (Fig. 5C) revealed statistically significant differences in the expression of JAK2 and NGF between the NF and DCM groups. These data indicate that NGF is highly expressed in the NF group, whereas JAK2 is predominantly expressed in the DCM group. The RT-qPCR expression trends of the hub genes are consistent with the preceding bioinformatics analysis. Furthermore, the ROC curve for the hub genes in distinguishing between DCM and NF samples is presented in Fig. 5D, with the AUC values for VCL, ABCB1, JAK2, KDR, and NGF being 76.4, 80.1, 68.2, 78.1, and 71.8, respectively, and ABCB1 demonstrating the highest accuracy (AUC = 80.1).

### Drug susceptibility analysis of hub gene

**Fig. 6:**
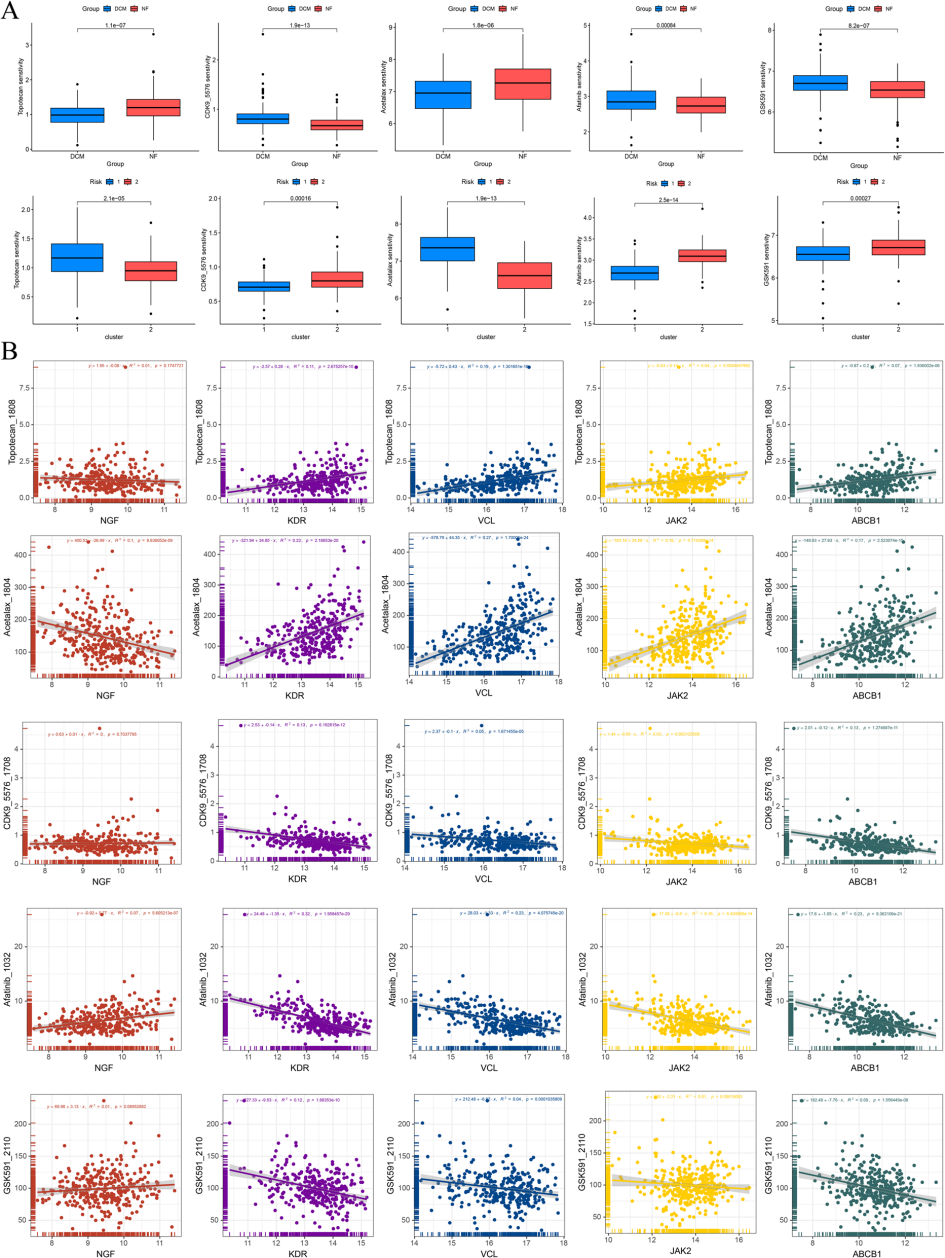
Drug susceptibility analysis of key genes. **A** Difference in drug susceptibility between DCM and NF group. **B** Drug susceptibility differences between cluster 1 and cluster 2. **C** Scatter plot of correlations between key genes and differential drugs

To identify small-molecule drugs significantly correlated with hub gene expression, a model was trained on the GDSC dataset, resulting in the identification of five key compounds: Topotecan, CDK9_5576, Acetalax, Afatinib, and GSK591. For each drug, differences in IC50 values between the NF and DCM groups were analyzed, as shown in Fig. 6A. The analysis revealed that CDK9_5576, Afatinib, and GSK591 exhibited higher IC50 values in the DCM group, whereas Topotecan and Acetalax demonstrated higher IC50 values in the NF group. Moreover, when comparing different clusters, Topotecan and Acetalax had higher IC50 values in cluster 1, while CDK9_5576, Afatinib, and GSK591 showed higher IC50 values in cluster 2. The correlation between hub gene expression and drug IC50 values is illustrated in Fig. 6B. Specifically, VCL, ABCB1, JAK2, and KDR were positively correlated with the IC50 values of Topotecan and Acetalax, whereas NGF was negatively correlated with the IC50 of CDK9_5576. Additionally, VCL, ABCB1, JAK2, and KDR exhibited negative correlations with the IC50 of Afatinib, while NGF showed a positive correlation. Furthermore, VCL, ABCB1, JAK2, and KDR were found to be negatively correlated with the IC50 of GSK591.

### Single cell location of hub gene

**Fig. 7:**
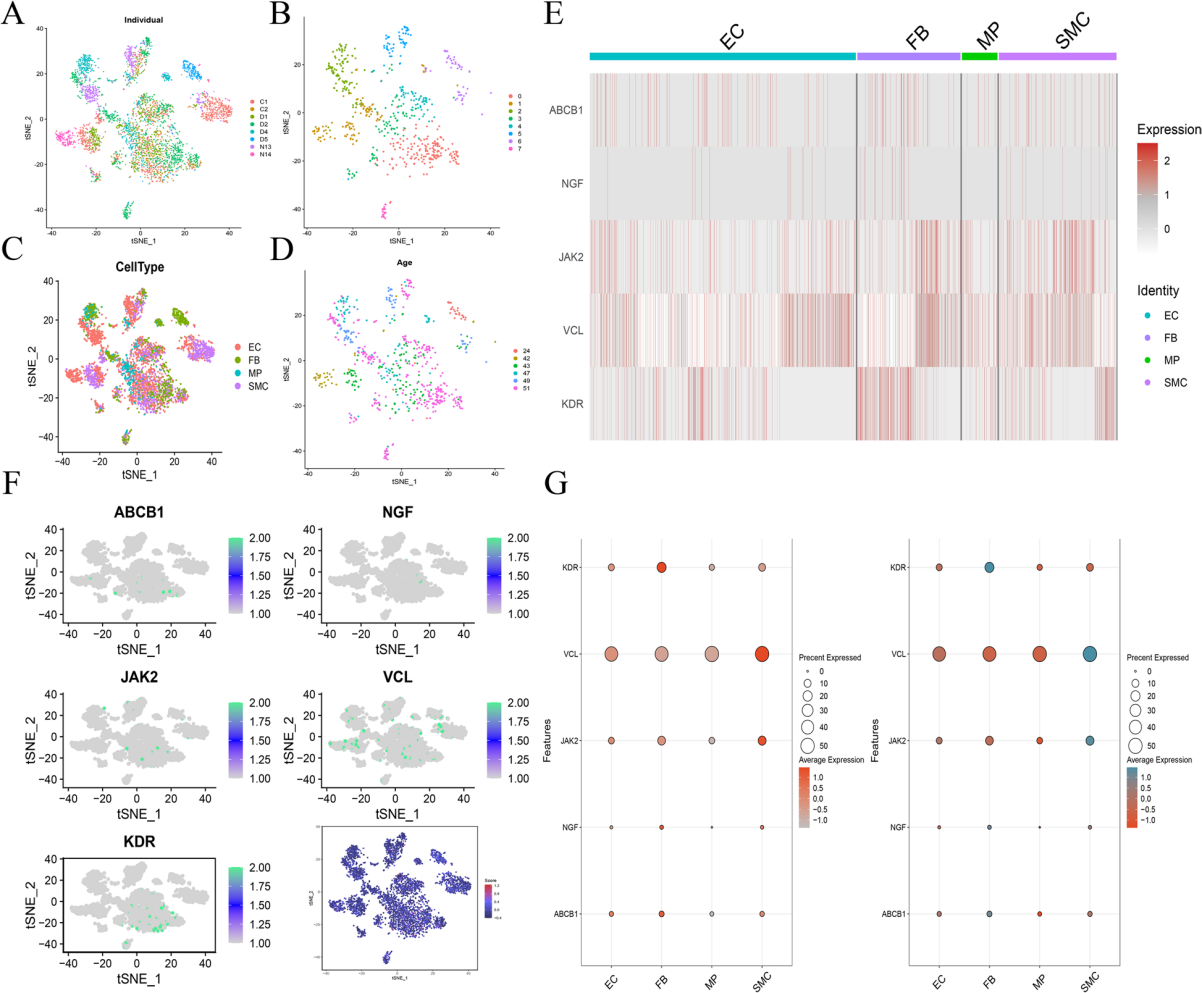
Single cell localisation of key genes in GSE121893 dataset. **A** UMAP results for different samples of single cells. **B** UMAP maps of cells from different clusters. **C** UMAP map of cell type annotation. **D** UMAP of cells at different ages. **E** Heat maps of key gene expression in different cell types. **F** Bubble plot of key gene expression in different cell types

The single-cell data from the GSE121893 dataset were integrated. The resulting UMAP visualization, shown in Fig. 7A, demonstrates that the majority of batch effects among the samples were effectively eliminated after integration. The cell groupings, illustrated in Fig. 7B, reveal eight distinct clusters. The cell annotation information was derived from the original dataset, as presented in Fig. 7C, yielding four distinct cell types: EC, FB, MR, and SMC. Considering that the samples span different age groups, the corresponding UMAP results are displayed in Fig. 7D. Figure 7E, F, and G depict the expression localization of the hub genes across different cell types, indicating that VCL is predominantly expressed in SMC cells of myocardial tissue, KDR in FB cells, JAK2 mainly in SMC cells, and ABCB1 primarily in FB cells, while NGF exhibits such low expression that its precise cellular origin is difficult to ascertain.

## Discussion

Dilated cardiomyopathy (DCM) represents a prototypical form of systolic heart failure (HF), characterized by cardiac dilation and a reduction in left ventricular ejection fraction (LVEF) [31, 32]. In recent decades, significant advances in techniques such as electrocardiography, advanced magnetic resonance imaging, and computed tomography have facilitated major breakthroughs in understanding its pathogenesis [1]. More recently, the application of various omics approaches, including metabolic analyses, has provided novel opportunities to identify prognostic and diagnostic biomarkers for DCM and to further elucidate the underlying mechanisms of the disease [33]. In particular, mitochondrial oxidative stress (ROS)-related functions have been demonstrated to compromise myocardial cell membranes and activate relevant signaling pathways, thereby exacerbating myocardial inflammation and contributing to the onset and progression of DCM [13].

This study employed bioinformatics methods to comprehensively investigate the potential mechanisms and diagnostic value of ROS in DCM. By analyzing multiple datasets, we screened for valuable biomolecules associated with DCM, including differential genes between NF and DCM samples as well as heterogeneous differential genes among DCM patients. In total, 64 genes were identified. Functional enrichment analysis revealed that these genes were predominantly associated with pathways related to cholesterol metabolism, lipid metabolism, and programmed cell death. Subsequently, through a protein–protein interaction network combined with LASSO and random forest models, five potential biomarkers—VCL, ABCB1, JAK2, KDR, and NGF—were ultimately identified. Immunoinfiltration analysis demonstrated notable co-expression between the hub genes and immune cells, including monocytes, CD8⁺ T cells, resting NK cells, and regulatory T cells, suggesting that these hub genes may modulate immune cell infiltration in DCM patients. The differential expression patterns of VCL, ABCB1, KDR, and NGF in the NF group, and of JAK2 in the DCM group, were further confirmed by RT-qPCR. Additionally, drug sensitivity analysis revealed that Topotecan, CDK9_5576, Acetalax, Afatinib, and GSK591 could directly influence the expression of these hub genes, potentially aiding clinicians in selecting appropriate therapeutic agents to improve patient prognosis. We also observed that VCL was highly expressed in EC, FB, MP, and SMC cells—particularly in SMC—while KDR was predominantly expressed in FB cells.

Furthermore, in addition to the five hub genes identified in this study, previous research has demonstrated that variations in VCL are significantly associated with the onset and progression of DCM, potentially contributing to early-onset DCM. ABCB1, a member of the ATP-binding cassette protein superfamily involved in the energy-dependent transport of various substrates across cellular membranes, may exhibit dysfunction that compromises drug efficacy and biological function, thereby adversely affecting the prognosis of DCM patients [34]. Moreover, significantly lower levels of KDR-positive cells in ICM patients compared to those with DCM [35] indicate heterogeneous expression of KDR between DCM and ICM patients, and signal transduction via gp130 and the JAK-STAT pathway is markedly altered in DCM. Furthermore, reduced tyrosine phosphorylation of JAK2 in the context of increased gp130 phosphorylation suggests impaired downstream activation of this critical pathway in DCM [36], representing a novel target for miR-98-5p. In addition, antithrombin-98-5p has been shown to alleviate microvascular dysfunction and enhance the expression of NGF and TRPV1 in a rat myocardial ischemia/reperfusion model [37].

These five genes play an important role in the diagnosis and treatment of DCM. On the one hand, we found that the five genes exhibited robust diagnostic power, with AUC values of 76.4, 80.1, 68.2, 78.1, and 71.8 for VCL, ABCB1, JAK2, KDR, and NGF in the NF and DCM groups, respectively, with all values exceeding 65%. We employed both the random forest and LASSO model algorithms and determined that these genes possess significant variable importance under both approaches. In addition, qPCR results further confirmed that the differences in gene expression between the DCM and NF groups were statistically significant, particularly for JAK2 and NGF. On the other hand, there is a strong correlation between these genes and immune cells. For example, NGF shows a significant positive correlation with NK cells, CD8⁺ T cells, and monocyte infiltration, suggesting that their high expression may play a therapeutic role by modulating the immune microenvironment of patients with DCM. Nevertheless, the following limitations remain: (1) The study primarily relied on bioinformatics analyses and transcriptome data from public databases, which may limit both the comprehensiveness of the data and the depth of experimental verification; (2) Although five key genes were identified through protein–protein interaction analyses and machine learning methods, the precise mechanisms by which these genes act in DCM remain to be fully verified through further experimentation; (3) Despite the collection of blood samples from DCM patients for validation, the sample size may be insufficient to represent the entire DCM patient population, potentially affecting the universality and reproducibility of the results; (4) While we have identified pharmaceutical agents that may influence the expression of the hub genes, the clinical efficacy and safety of these agents have not yet been evaluated in large-scale clinical trials. It is hoped that future studies will validate the expression patterns of these key genes and demonstrate their potential utility in diagnosing DCM in a larger patient population. Furthermore, additional experimental studies, including animal models and cellular experiments, will help elucidate the precise roles of these genes in the pathogenesis of DCM.

## Conclusion

In this study, bioinformatics analyses and multiomics techniques were employed to elucidate the molecular mechanisms underlying dilated cardiomyopathy (DCM) and to identify five potential biomarkers: VCL, ABCB1, JAK2, KDR, and NGF. These markers play a pivotal role in the pathogenesis of DCM and are closely associated with immune cell infiltration. The study also revealed that these genes exhibit specific expression patterns across different cell types, offering novel insights for cell-specific treatment approaches in DCM. Furthermore, these biomarkers demonstrated robust diagnostic efficacy, with AUC values exceeding 70%, thereby providing new therapeutic targets and diagnostic tools for the early and precise treatment of DCM.

## Data Availability

No datasets were generated or analysed during the current study.
